# Supplementary material for: Corrosion Resistance in Artificial Perspiration of Cr-Based Decorative Coatings
Source: Nanomaterials (Basel). 2023 Aug 15;13(16):2346. doi: 10.3390/nano13162346 (PMC10459534; doi:10.3390/nano13162346)
Supplement: Supplementary file 1 [file nanomaterials-13-02346-s001.zip › nanomaterials-2531565-supplementary.pdf]

Supplementary Figure S1

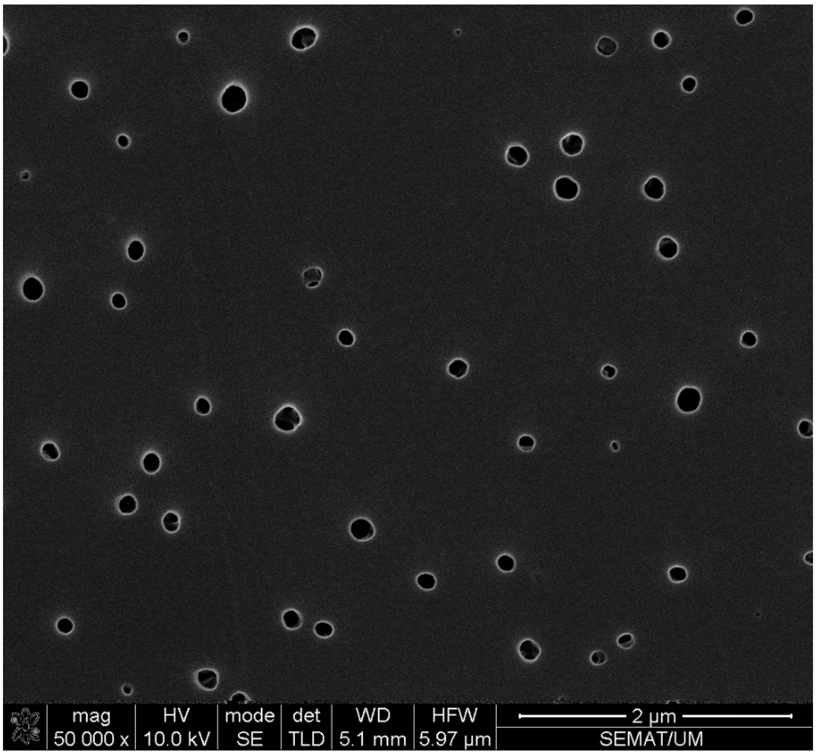

Figure S1 - SEM micrographs of the epoxy surface.

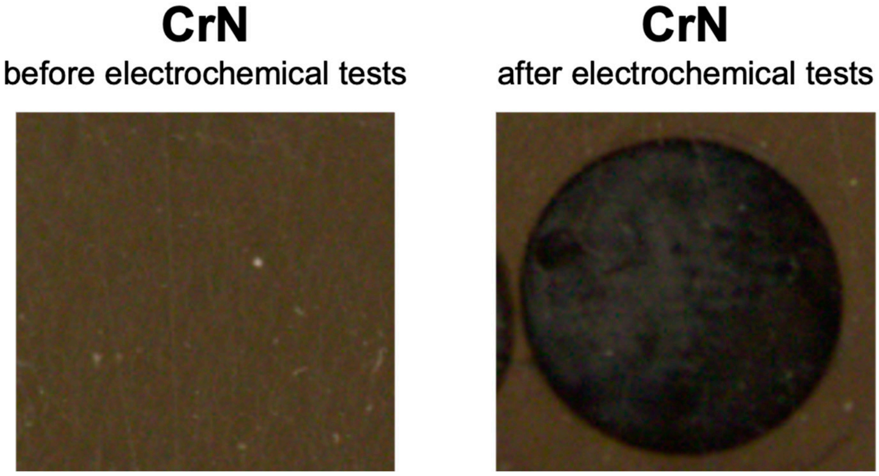

Figure S2 - By naked eye visualization of CrN sample before and after electrochemical testing
